# Vitamin D in Irritable Bowel Syndrome: Exploring Its Role in Symptom Relief and Pathophysiology

**DOI:** 10.3390/nu17061028

**Published:** 2025-03-14

**Authors:** Ioanna Aggeletopoulou, Georgios Geramoutsos, Ploutarchos Pastras, Christos Triantos

**Affiliations:** Division of Gastroenterology, Department of Internal Medicine, University of Patras, 26504 Patras, Greece; giorgosgeramoutsos@gmail.com (G.G.); ploutarchosp96@gmail.com (P.P.); chtriantos@upatras.gr (C.T.)

**Keywords:** irritable bowel syndrome, IBS, vitamin D, gut microbiome, symptom severity, quality of life, pathophysiology

## Abstract

Irritable Bowel Syndrome (IBS) is a chronic functional gastrointestinal disorder. Despite its common occurrence, the pathophysiology of IBS remains not fully understood. Emerging evidence suggests that IBS is a multifactorial condition characterized by low-grade inflammation, immune system activation, impaired gut permeability, intestinal hypersensitivity, and alterations in intestinal microbiota. Recent data have highlighted the potential role of vitamin D in modulating these underlying mechanisms. Vitamin D is known to influence various cellular processes, including the regulation of the gut microbiome, immune response modulation, and anti-inflammatory effects, which may alleviate the altered gut function observed in IBS. Research indicates that individuals with IBS often have lower levels of vitamin D compared to healthy controls, suggesting a possible link between vitamin D deficiency and IBS. Vitamin D supplementation has been associated with improvements in IBS symptoms, such as bloating, flatulence, abdominal pain, constipation, and overall quality of life. The mechanisms by which vitamin D exerts these effects may involve direct or indirect modulation of immune responses, the production of antimicrobial peptides, and the regulation of gene expression related to serotonergic metabolism. Despite these promising findings, the exact pathways through which vitamin D affects IBS pathophysiology remain unclear. The aim of this review is to outline the current knowledge and evidence regarding these mechanisms, as well as the therapeutic potential of vitamin D supplementation in IBS patients. Exploring the connection between vitamin D and IBS may pave the way for innovative interventions, enhancing both management strategies and the quality of life for those affected by the disorder.

## 1. Introduction

Irritable bowel syndrome (IBS) is one of the most prevalent functional gastrointestinal disorders and a leading cause of gastroenterology consultations in Western countries [1]. Its prevalence ranges from 10% to 25% of the general population, with major regional variations; South Asia reports the lowest prevalence at 7.0%, while South America has the highest at 21.0% [2]. IBS symptoms primarily include abdominal pain or discomfort, bloating, and changes in bowel habits, including diarrhea and/or constipation [3,4]. IBS is categorized into four subtypes according to predominant bowel habits: diarrhea-predominant (IBS-D), constipation-predominant (IBS-C), mixed-type (IBS-M), and unclassified (IBS-U) [5,6]. The IBS-U subtype applies to cases where stool patterns do not consistently fit the criteria for the other classifications, presenting a challenge for accurate diagnosis [5,6].

**Figure 1:**
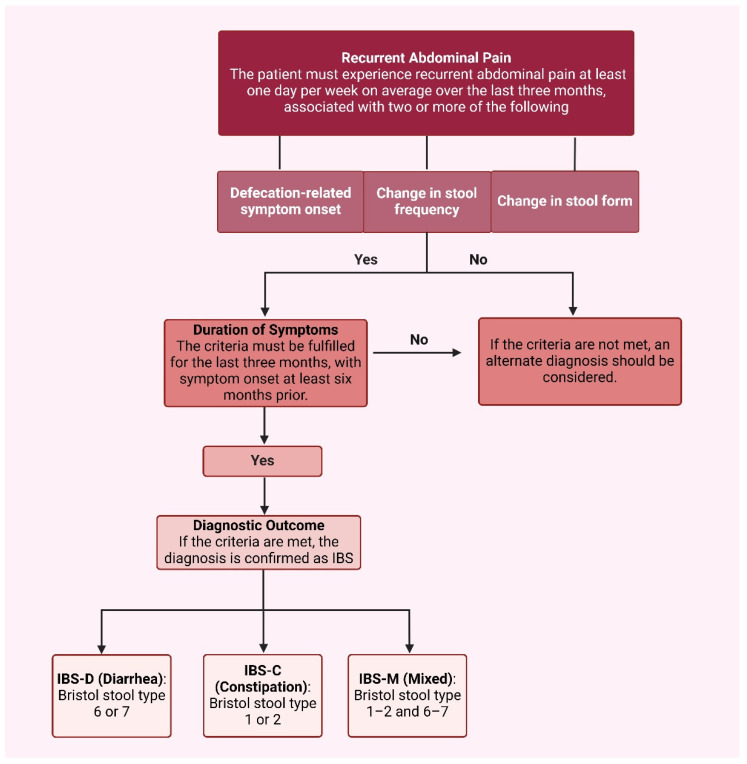
IBS diagnostic criteria. Created with BioRender.com (accessed on 5 March 2025).

Due to the lack of a definitive diagnostic test or biomarker for IBS, symptom-based diagnostic criteria were developed through expert consensus to establish a consistent diagnostic approach and reduce unnecessary testing. The Rome criteria serve as the primary framework for this purpose. These criteria have undergone multiple revisions, with the Rome III version implemented in 2006 [7], and the Rome IV criteria introduced in 2016 [3]. Key modifications in the Rome IV criteria include the removal of abdominal discomfort from the definition and a heightened frequency requirement for abdominal pain, changing from at least three days per month to at least one day per week [3]. As a result, the Rome IV criteria are more stringent compared to their predecessor, leading to a reduction in the number of self-diagnosed IBS cases that meet the formal classification (Figure 1).

The pathophysiology of IBS is complicated and multifactorial, involving a combination of genetic, epigenetic, environmental, and physiological factors [1,8]. While its exact mechanisms remain unclear, IBS is widely recognized as a disorder arising from dysregulation of the gut–brain axis (BGA), involving disruptions in the enteric, autonomic, and central nervous systems, or their intricate interactions [1,8]. Several factors are implicated in the pathogenesis of IBS, including gastrointestinal motility dysfunction, post-infectious immune reactivity, gut microbiota dysbiosis, small intestinal bacterial overgrowth (SIBO), carbohydrate malabsorption, food sensitivities, and low-grade intestinal inflammation [9]. In addition, stress-related nervous and endocrine system activity, along with immune dysregulation, may predispose individuals to IBS [10]. Factors such as diet, exposure to toxins, stressful life events, persistent infections, and changes in the gut microbiome are thought to be significant contributors to the development of symptoms [10].

Individuals who do meet the criteria for IBS experience more intense symptoms and present a higher prevalence of psychological comorbidities [11,12]. In particular, these patients frequently exhibit a critical decrease in health-related quality of life (QoL) [13], along with elevated levels of somatization [14], an increase in psychological comorbidities, including depression and suicidal thoughts [15], along with a decrease in work productivity [16].

IBS remains a challenging condition to manage, as a significant proportion of patients do not respond adequately to conventional treatments [17]. This has led approximately one-third of individuals with IBS to explore alternative therapies [18]. Among the factors being investigated for their potential role in IBS, vitamin D has gained considerable attention. Vitamin D deficiency is considered a very common challenge, affecting 30–50% of the global population across various demographic groups [19,20], and is linked to numerous chronic conditions, including cardiovascular disease [21], cancer [22], insulin resistance [23], liver disease [24,25] and gastrointestinal disorders [26,27]. Given its crucial role in calcium and phosphorus metabolism, immune regulation, and anti-inflammatory processes [28], vitamin D is pivotal for maintaining the integrity of mucosal surfaces, including the intestinal barrier [29]. Thus, research has been focused on the potential link to IBS, as its deficiency can lead to mucosal damage and gastrointestinal symptoms [30]. Overall, these findings underscore the potential role of vitamin D in the pathophysiology and management of IBS. This review aims to describe the potential role of vitamin D in the pathophysiology and management of IBS by examining the mechanisms through which it may influence the disorder, evaluating the evidence supporting its therapeutic use, and assessing its impact on symptom severity and QoL.

## 2. Vitamin D Uptake, Metabolism, Physiology, and the Role of Vitamin D Receptor

Vitamin D, a fat-soluble nutrient, plays a vital role in numerous physiological processes throughout the human body [31]. Beyond its well-known function as a prohormone, vitamin D is essential for more than just regulating calcium and phosphorus metabolism and supporting bone remodeling [31,32,33]. It actively contributes to the modulation of immune responses by enhancing the antimicrobial activity of monocytes and macrophages, as well as by regulating inflammation [33,34,35]. In addition, when the active form of vitamin D, calcitriol, interacts with the vitamin D receptor (VDR) present in various tissues, it influences the expression of genes responsible for cell growth, differentiation, and immune regulation [33,34,35].

The physiological effects of vitamin D are regulated through a series of metabolic processes taking place mainly in the skin, kidneys, and liver. The two main forms of vitamin D, vitamin D2 (ergocalciferol) and vitamin D3 (cholecalciferol), differ structurally and functionally [36]. Vitamin D2 is derived from plant sources, particularly through the ultraviolet irradiation of ergosterol found in fungi and yeast sterol. This form contains a double bond between carbons 22 and 23, along with a methyl group at carbon 24 [36]. Conversely, vitamin D3 is produced in the skin’s epidermis when exposed to UVB radiation (290–320 nm) or obtained from animal sources [37]. It lacks the features present in vitamin D2, making it more bioavailable and effective at maintaining vitamin D levels in the body [36,37].

When exposed to UVB radiation, 7-dehydrocholesterol in the skin is converted into pre-vitamin D3, which then undergoes thermal isomerization to produce vitamin D3 [36]. Vitamin D can also be acquired through dietary sources; in these cases, its absorption, which depends on dietary fat intake due to its fat-soluble nature, occurs in the small intestine, where it is incorporated into micelles and absorbed into enterocytes [38]. After being absorbed by enterocytes, vitamin D binds to vitamin D-binding protein (DBP) and is then transported to various tissues, including the liver. In the liver, it is converted by 25-hydroxylase (CYP2R1) into 25-hydroxyvitamin D [25(OH)D], which is the main circulating form [34,35,39]. In the kidneys, primarily in the proximal tubule, 1α-hydroxylase (CYP27B1) further hydroxylates 25(OH)D to form 1,25-dihydroxyvitamin D3 [1,25(OH)2D3] or calcitriol, the biologically active form [34,35,39]. Calcitriol binds to DBP and is delivered to target tissues, such as the intestine, kidneys, and bones, where it regulates the homeostasis of calcium and phosphate [38]. Vitamin D metabolism is tightly controlled, with 25(OH)D 24-hydroxylase (CYP24A1) deactivating calcidiol and calcitriol into inactive forms for excretion [40]. Although the active form, 1,25(OH)2D3, drives physiological effects, 25(OH)D is measured to assess vitamin D status due to its longer half-life and stable blood levels [41,42]. Vitamin D metabolism is regulated by factors such as parathyroid hormone (PTH), calcium, phosphorus, and fibroblast growth factor (FGF) [41,42].

Calcitriol exerts its function by binding to VDR, a nuclear receptor encoded by the *VDR* gene and present in most human cells [43,44]. The VDR regulates about 3–5% of the human genome, influencing numerous biological processes, including immune regulation and calcium homeostasis [45]. Structurally, VDR’s ligand-binding domain interacts with 1,25(OH)2D3, while its DNA-binding domain binds to vitamin D response elements (VDREs) in target gene promoters. Following activation, VDR creates a complex with the retinoid X receptor (RXR), driving transcriptional changes that regulate target gene expression [46].

In addition to genomic pathways, non-genomic pathways allow calcitriol to interact with membrane-bound VDR (1,25D-MARRS) [47]. This triggers rapid intracellular signaling changes through pathways such as mitogen-activated protein kinase (MAPK), phospholipase A2 (PLA2), phospholipase C (PLC), and phosphatidylinositol-3 kinase (PI3K), affecting diverse cellular functions [47]. These mechanisms underscore vitamin D’s broad biological roles, spanning calcium absorption, immune modulation, and disease mitigation.

Understanding the physiological role of vitamin D metabolism and the VDR provides a basis for exploring its potential impact on conditions including IBS. Vitamin D, through its active form, regulates gene expression and immune responses by binding to VDR, which is expressed in various tissues, including the gastrointestinal tract. This interaction suggests that vitamin D may influence gut homeostasis, immune modulation, and inflammation, key factors implicated in IBS pathophysiology. Given this mechanistic link, research has turned to meta-analyses to assess the clinical evidence supporting the role of vitamin D in IBS. These studies aim to determine whether vitamin D supplementation could offer therapeutic benefits, potentially addressing the underlying dysregulation observed in IBS patients.

## 3. Data from Meta-Analyses on the Role of Vitamin D on IBS

Vitamin D has been increasingly studied for its potential role in IBS, a disorder that affects gut function and significantly impacts patients’ QoL. Several meta-analyses have investigated whether vitamin D supplementation can influence key IBS-related outcomes, including symptom severity, QoL, mental health (anxiety and depression), and serum vitamin D levels. In this section, findings from meta-analyses are thoroughly examined, providing an evidence-based evaluation of the role of vitamin D supplementation in IBS management (Table 1).

### 3.1. Effect of Vitamin D Supplementation on Severity of Symptoms

The effect of vitamin D supplementation on IBS symptom severity has been extensively examined, with conflicting findings across meta-analyses. A recent meta-analysis presented that vitamin D significantly ameliorated symptom severity as measured by the Irritable Bowel Severity Scoring Scale (IBS-SSS), with an SMD of −0.77 (95% CI: −1.47 to −0.07, *p* = 0.04) [50]. However, the results exhibited high heterogeneity (I^2^ = 91%), reflecting variability among included studies [50]. Sensitivity analysis revealed that removing studies with a moderate-to-high risk of bias diminished the statistical significance of improvements in IBS symptoms, highlighting limitations related to study quality [50]. Additionally, subgroup analysis showed that studies using the Rome III criteria demonstrated a significant improvement in IBS symptom severity, whereas those using Rome IV criteria did not, potentially due to the inclusion of more severe IBS cases in the latter [50]. Huang et al. provided further strong evidence that vitamin D significantly reduced IBS symptom severity, with a WMD of −55.55 (95% CI: −70.22 to −40.87, I^2^ = 53.7%) after intervention, compared to a non-significant difference before intervention (WMD: −3.17, 95% CI: −18.15 to 11.81, I^2^ = 0.0%) [51]. Participants who received vitamin D supplementation experienced marked improvement in IBS-SSS, with a WMD of −84.21 (95% CI: −111.38 to −57.05, I^2^ = 73.2%), compared to −28.29 (95% CI: −49.95 to −6.62, I^2^ = 46.6%) in the placebo group [51].

Conversely, Abuelazm et al. displayed that vitamin D had no considerable impact on symptom severity, with an MD of −45.82 (95% CI: −93.62 to 1.98, *p* = 0.06) [48]. Sensitivity analysis suggested a slight improvement after removing certain studies, but the overall evidence remained inconclusive [48]. In line with these results, the meta-analysis by Bin et al. exhibited that vitamin D supplementation did not significantly affect symptom severity, with an SMD of −0.43 (95% CI: −0.89 to 0.03). Similarly, Cara et al. found that vitamin D supplementation did not lead to a significant reduction in IBS symptom severity, with a pooled MD of −25.89 (95% CI: −55.26 to 3.48, I^2^ = 92.8%).

The systematic review by Veraza et al. demonstrated that vitamin D deficiency may be linked to IBS symptom severity, as observational studies consistently reported lower serum vitamin D levels in patients with IBS [53]. However, despite the association, the review found no clear evidence that vitamin D intake levels significantly differed between IBS patients and non-IBS controls [53].

Overall, while some studies support a beneficial role of vitamin D on IBS symptom severity, others report no significant effect, indicating the need for larger, high-quality RCTs to clarify its therapeutic role.

### 3.2. Effect of Vitamin D Supplementation on Quality of Life (QoL)

Several meta-analyses assessed the effect of vitamin D supplementation on QoL in patients with IBS, with varying results. Abuelazm et al. demonstrated that vitamin D significantly ameliorated the IBS-QoL in patients with IBS, with a mean difference (MD) of 6.19 (95% CI: 0.35 to 12.03, *p* = 0.04) [48]. While heterogeneity was observed, excluding one particular study [54] resolved the issue and strengthened the findings, yielding an MD of 3.26 (95% CI: 2.14 to 4.39, *p* = 0.00001) over placebo [48]. Similarly, Huang et al. demonstrated that vitamin D supplementation was more effective than placebo in ameliorating IBS-QoL, with a weighted mean difference (WMD) of 14.98 (95% CI: 12.06 to 17.90, I^2^ = 0.0%), whereas the placebo group exhibited a WMD of 6.55 (95% CI: −2.23 to 15.33, I^2^ = 82.7%) [51]. The meta-analysis by Bin et al. agreed that vitamin D significantly improved QoL scores, with an SMD of 0.65 (95% CI: 0.14 to 1.15) [49]. Additionally, Cara et al. reported that QoL scores improved significantly in populations with vitamin D deficiency at baseline following vitamin D supplementation, with an MD of 3.19 (95% CI: 2.14 to 4.24, I^2^ = 0.0%) [52].

Conversely, Chong et al. showed that vitamin D did not significantly improve IBS-QoL, with a standardized mean difference (SMD) of 0.54 (95% CI: −0.34 to 1.41, *p* = 0.15) [50]. The results were affected by substantial heterogeneity (I^2^ = 87% for QoL), reflecting variability among the included studies [50]. Sensitivity analyses suggested that excluding studies with a moderate-to-high risk of bias reduced the statistical significance of IBS symptom improvement, indicating potential limitations in study quality [50]. Notably, Abuelazm et al. in their commentary expressed concerns about the meta-analysis by Chong et al., to the effect that the methodological issues identified could lead to misleading results [55]. The systematic review by Veraza et al. revealed that some randomized controlled trials (RCTs) in vitamin D-deficient populations demonstrated improvements in QoL following vitamin D supplementation [53]. However, other studies found no notable variation in QoL between the vitamin D-treated and placebo groups, despite similar dosing regimens [53].

While most meta-analyses support a beneficial role of vitamin D supplementation on QoL in IBS patients, others suggest limited or inconsistent effects, highlighting the need for further research to determine its therapeutic potential and optimal dosing strategies.

### 3.3. Effect of Vitamin D Supplementation on Other Outcomes

Vitamin D dosing and testing are important considerations in the management of IBS patients. It is recommended to assess vitamin D levels, particularly in those with comorbid conditions or signs of deficiency. In cases of low vitamin D, supplementation typically ranges from 2000 IU/day to 50,000 IU/biweekly, though higher doses may be necessary for patients with significant deficiency [50]. Dosing should be individualized, and regular monitoring of vitamin D levels is essential to ensure optimal therapeutic outcomes.

Beyond symptom severity and QoL, several meta-analyses explored the impact of vitamin D supplementation on serum vitamin D levels and deficiency risk in IBS patients. The meta-analysis by Bin et al. confirmed that IBS patients generally have lower serum vitamin D levels compared to healthy individuals [49]. Additionally, IBS patients were found to be at a higher risk of vitamin D deficiency, with a relative risk (RR) of 1.78 (95% CI: 1.45 to 2.12) [49]. Cara et al. found that vitamin D supplements substantially raised serum 25(OH)D levels compared to placebo, with a pooled MD of 20.33 ng/mL (95% CI: 12.91 to 27.74, I^2^ = 97.9%) [52]. In line with this, Abuelazm et al. demonstrated that vitamin D supplementation effectively increased serum 25(OH)D levels compared to placebo, with an MD of 25.2 (95% CI [18.41, 31.98], *p* = 0.00001), confirming its efficacy in raising vitamin D levels in IBS patients [48]. The meta-analysis by Veraza et al. evaluated the impact of nutrient intake and diet on various IBS-related outcomes [53]. Focusing on vitamin D, the meta-analysis exhibited that vitamin D intake was suboptimal in IBS patients, failing to meet the recommended dietary reference values (DRV) [53]. However, the analysis did not provide clear evidence of a major difference in vitamin D intake between IBS patients and non-IBS controls [53]. Despite this, observational studies have consistently demonstrated lower vitamin D levels in IBS patients, suggesting a potential association [53]. Głąbska et al. systematically reviewed the role of vitamin D supplementation on mental health in patients with IBS [56]. The results revealed that vitamin D supplementation had a positive influence on mental health in IBS patients, particularly in improving QoL, anxiety, and depression scores [56]. Notably, all studies assessing anxiety and depression reported beneficial effects, while most studies evaluating QoL confirmed improvements [56]. However, certain studies did not find significant effects, highlighting variability in outcomes [56]. Different supplementation regimens and dosages were used, but the overall trend suggested a consistent positive influence of vitamin D on mental well-being [56]. Despite these findings, limitations included a small number of studies, potential heterogeneity in study designs, and a lack of trials evaluating other mental health outcomes such as stress, self-esteem, and suicidal ideation [56].

The variability in findings suggests that vitamin D status, rather than intake alone, may be a more critical factor in IBS pathophysiology and symptom severity. While some studies demonstrate that IBS patients have lower serum vitamin D levels, others do not find a great difference in dietary vitamin D intake between IBS patients and healthy controls. This indicates that insufficient vitamin D absorption, metabolism, or utilization, rather than inadequate intake, may contribute to IBS pathophysiology. Additionally, RCTs in vitamin D-deficient populations have shown that correcting deficiency through supplementation can improve IBS symptom severity, suggesting that the baseline vitamin D status of patients influences treatment outcomes. In contrast, studies including patients with normal or mildly low vitamin D levels often report no significant effect of supplementation, further emphasizing that deficiency correction, rather than additional intake beyond sufficient levels, may be the key factor.

## 4. Role of Vitamin D on IBS Pathophysiology

The high prevalence of vitamin D deficiency among individuals with IBS has been reported in many studies, indicating a possible relation between vitamin D status and the development or exacerbation of IBS symptoms, particularly in relation to inflammation, a key factor in IBS pathophysiology. These findings have encouraged further exploration of vitamin D supplementation as a therapeutic approach to improve IBS symptoms. In parallel, growing evidence suggests that vitamin D may influence multiple mechanisms involved in IBS, including gut microbiota modulation [57], antimicrobial peptide release [58], regulation of intestinal permeability [59], inflammatory and immune responses [60,61] and gut–brain communication impairment [62]. Vitamin D suppresses Th1/Th17 cells while promoting T regulatory cells, which helps reduce intestinal inflammation, a key factor in IBS pathophysiology [63]. Vitamin D exhibits an immunomodulatory role by enhancing the secretion of antimicrobial peptides and regulating intestinal epithelial cell integrity [64]. Antimicrobial peptides are crucial for gut health, regulating microbiota, maintaining barrier integrity, and modulating immune responses [65]. In IBS, impaired gut barrier function and dysbiosis contribute to inflammation. AMPs like cathelicidins and defensins help control microbial balance and reduce pathogenic overgrowth [65]. Vitamin D enhances AMP production via the VDR, promoting gut health [66]. Sufficient vitamin D may restore intestinal barrier function, reduce inflammation, and rebalance the microbiota, potentially alleviating IBS symptoms.

### 4.1. Vitamin D in Modulating Gut Microbiome Composition and Its Potential Impact on IBS

The dynamic shifts in the microbiome, characterized by altering the composition of specific bacterial taxa in IBS, have been shown to significantly influence disease progression and pathology [67]. Beneficial species like Lactobacillus and Bifidobacterium have been reduced, while potentially harmful bacteria, such as Firmicutes and Proteobacteria, have increased [67]. Vitamin D has been suggested as a modulator of the gut microbiome, with some studies suggesting it may contribute to microbial diversity and composition. Research indicates that vitamin D supplementation can alter the gut microbiota increasing the prevalence of beneficial bacteria and decreasing the microbial composition of harmful types [68,69]. This modulation has led to the hypothesis that vitamin D contributes to improved gut health and may alleviate IBS symptoms by influencing gut microbiota composition. However, conclusive data confirming this relationship remain scarce. In contrast, a retrospective case-control study investigating the association between plasma vitamin D levels and gut microbiome composition in adult women diagnosed with IBS compared to healthy controls found no significant difference in vitamin D levels between the two groups [70]. Statistical analysis further revealed no significant association between vitamin D status and the likelihood of IBS [70]. Additionally, no correlation was observed between plasma 25(OH)D levels and gut microbiome diversity metrics, including richness, Shannon index, Simpson index, or the relative abundances of key bacterial taxa such as Bifidobacterium and Lactobacillus [70]. These data indicate that the proposed link between vitamin D status and gut microbiome composition in IBS remains inconclusive. Therefore, further well-designed studies are essential to clarify whether vitamin D plays a meaningful role in shaping gut microbiome composition in IBS patients.

### 4.2. Serotonergic Signaling in IBS and the Role of Vitamin D

The relationship between serotonergic metabolism and IBS is complex and not fully understood. However, selective serotonin reuptake inhibitors (SSRIs), commonly used to treat depression, have been observed to alleviate IBS symptoms, suggesting a significant role for serotonin in IBS pathophysiology [71].

Serotonin synthesis in the gastrointestinal tract is primarily regulated by the enzyme tryptophan hydroxylase (TPH), with TPH1 being the main isoform in this region. Research indicates that vitamin D, particularly its active form calcitriol (1,25-dihydroxyvitamin D), can modulate the expression of TPH1 and the serotonin transporter (SERT), influencing serotonin production [72,73]. Additionally, vitamin D plays a crucial role in the nervous system, with VDR expressed throughout the brain, influencing neurotransmitter levels and serotonin synthesis, thereby affecting gut motility and sensitivity [72].

The active form of vitamin D upregulates neurotrophins, promoting the survival and differentiation of nerve cells [74], and along with the VDR modulates the epithelial barrier function in the gut [75]. Vitamin D deficiency could potentially impact bowel function by disrupting these processes, contributing to IBS pathophysiology.

Gene expression and variations in vitamin D and serotonin pathways in individuals with IBS have been assessed. Studies show that IBS patients have lower levels of 25(OH)D compared to non-IBS controls, as well as reduced expression of TPH1, the enzyme responsible for the rate-limiting step in serotonin synthesis in gut enterochromaffin cells [76]. These findings suggest that vitamin D deficiency and dysregulated serotonin production may contribute to the development and progression of IBS. Additional studies have revealed that vitamin D can upregulate the expression of monoamine oxidase (MAO), the enzyme responsible for degrading serotonin into 5-hydroxyindoleacetic acid, influencing serotonin levels [77,78]. In addition, vitamin D has been found to enhance the expression of SERT in brain neurons, affecting serotonin reuptake [77].

Further research has suggested that vitamin D regulates tryptophan hydroxylase enzymes, promoting the conversion of tryptophan to serotonin in the brain by increasing TPH2 activity, while decreasing TPH1 activity in non-brain tissues [72,79]. This modulation suggests that vitamin D influences serotonin production across different body systems [72,79]. A randomized clinical trial assessed the effects of vitamin D3 supplementation in patients with IBS-D and found significant improvements in IBS severity scores, QoL, depression, and visceral sensitivity index scores compared to the placebo group [80]. However, no significant differences were found in abdominal bloating, anxiety, serum serotonin, 5-hydroxy-indole acetic acid, or the ratio of 5-HIAA/5-HT [80]. The authors noted that extending the intervention period or using more precise biochemical evaluations might have yielded statistically significant differences [80].

Grozić et al. analyzed gene expression in sigmoid colon mucosal samples from IBS patients and healthy controls [81]. They identified 858 differentially expressed genes, including 23 genes related to serotonin metabolism [81]. Notably, the study found that vitamin D could modulate the expression of four IBS-related genes (downregulation of TDRD6 and FLT4 and upregulation of SERT and TPH1), suggesting a connection between vitamin D levels and serotonin pathways in IBS pathophysiology [81]. These findings propose that specific gene expression patterns, particularly in serotonin metabolism, may serve as biomarkers for IBS and that vitamin D status could influence these molecular mechanisms, potentially alleviating symptoms [81].

**Figure 2:**
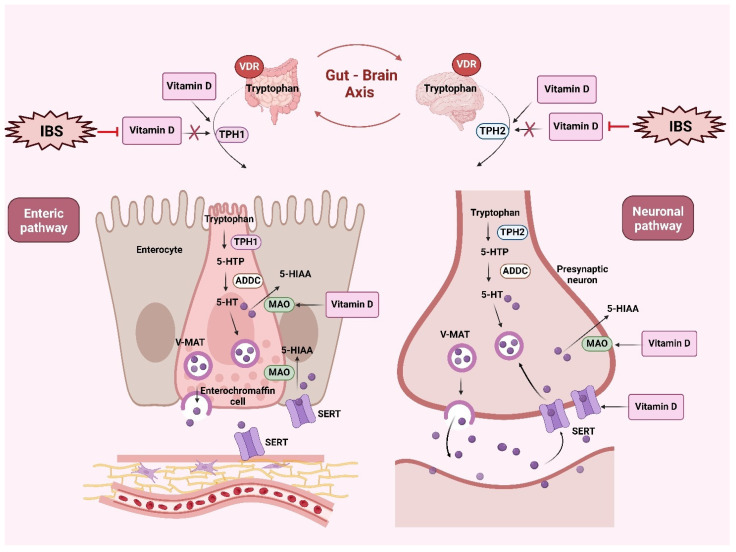
Serotonergic metabolism in Irritable Bowel Syndrome (IBS) and the modulatory role of vitamin D. Figure 2 illustrates the interplay between vitamin D and serotonergic signaling in the gut–brain axis, highlighting its implications in Irritable Bowel Syndrome (IBS). On the left, the enteric pathway is depicted, where tryptophan is converted to 5-hydroxytryptophan (5-HTP) by tryptophan hydroxylase 1 (TPH1) in enterochromaffin cells, and then to serotonin (5-HT) by aromatic L-amino acid decarboxylase (ADDC). Serotonin is stored in vesicles via the vesicular monoamine transporter (V-MAT) and released into the intestinal lumen and bloodstream, where it regulates gut motility and sensation. The serotonin transporter (SERT) facilitates serotonin reuptake, while excess serotonin is degraded to 5-hydroxyindoleacetic acid (5-HIAA) by monoamine oxidase (MAO). Vitamin D modulates this pathway by influencing TPH1 expression and regulating both SERT and MAO activity. Additionally, the activation of the vitamin D receptor (VDR) in the enteric pathway can upregulate TPH1 expression, enhancing serotonin synthesis from tryptophan. Conversely, vitamin D deficiency is potentially associated with downregulation of TPH1 and disruption of serotonin balance, exacerbating IBS symptoms. On the right, the neuronal pathway of the gut–brain axis is shown, where tryptophan is converted to serotonin through TPH2 and ADDC in presynaptic neurons. Serotonin is stored in vesicles via V-MAT, released into the synaptic cleft, and reabsorbed through SERT. Vitamin D, acting via the VDR, modulates TPH2 and SERT expression, thereby influencing serotonin turnover. Overall, the figure highlights how vitamin D, through its effects on TPH1, TPH2, SERT, and MAO, plays a crucial role in regulating serotonin availability, which affects gut motility, visceral sensitivity, and mood. Furthermore, in this figure we suggest that vitamin D deficiency may disrupt serotonergic signaling, exacerbating IBS symptoms and contributing to the gut–brain axis imbalance observed in IBS patients. Created with BioRender.com (accessed on 13 February 2025). Abbreviations: IBS, irritable bowel syndrome; VDR, vitamin D receptor; TPH1, tryptophan hydroxylase-1; 5-HT, serotonin; 5-HTP: 5-hydroxytryptophan; ADDC, aryldialkylamine *N*-acyltransferase; 5-HIAA: 5-hydroxyindoleacetic acid; MAO, monoamine oxidase; SERT, serotonin transporter; V-MAT, vesicular monoamine transporter.

Figure 2 illustrates the dual role of vitamin D in modulating serotonergic metabolism through the gut–brain axis in IBS pathophysiology, highlighting its regulatory effects on TPH isoforms, serotonin production, and reuptake processes within enterochromaffin cells and presynaptic neurons.

Further studies have suggested that vitamin D supplementation enhances vitamin D levels and QoL in IBS patients [82]. Notably, IBS-C patients showed the most responsiveness to vitamin D supplementation, with improvements across nearly all IBS symptoms [82]. This provides evidence for the hypothesis that vitamin D may increase TPH1 serotonin levels in the gut, potentially alleviating IBS symptoms [73].

### 4.3. Vitamin D and Its Role in Gut Health and the Gut–Brain Axis in IBS

The link between vitamin D and gastrointestinal health has been increasingly recognized, particularly in the context of IBS. Linsalata et al. investigated the connection between low serum vitamin D levels and altered intestinal barrier function in patients with IBS-D adhering to a long-term low-fermentable oligosaccharides, disaccharides, monosaccharides, and polyol (FODMAP) diet (LFD) [83]. The study demonstrated that LFD significantly improved vitamin D levels, reduced IBS-D symptoms, and enhanced intestinal barrier function, especially in patients with low baseline vitamin D levels [83]. Notably, these patients had significantly higher small intestinal permeability compared to those with normal vitamin D values, as evidenced by elevated lactulose excretion and La/Mannitol ratios [83]. Following LFD, small intestinal permeability improved in both groups, with low vitamin D patients showing a 54% reduction in lactulose excretion and a 31% decrease in the La/Ma ratio, reaching normal levels [83]. Furthermore, fecal zonulin, a marker of intestinal barrier dysfunction, was higher in low-vitamin D patients at baseline but decreased significantly after the LFD, especially in this cohort [83]. Other markers of gut barrier integrity, including serum zonulin, intestinal fatty acid-binding protein (I-FABP), and diamine oxidase (DAO) levels, also decreased following LFD, indicating improved intestinal function [83]. Inflammatory markers interleukin-6 (IL-6) and IL-8, and lipopolysaccharide (LPS) levels, indicative of bacterial translocation, decreased in low-vitamin D patients after the intervention, supporting the potential role of vitamin D in modulating inflammation and ameliorating gut health [83]. Regression analysis further suggested that vitamin D levels could be predicted by IBS-SSS scores and fecal zonulin levels, highlighting the relationship between vitamin D, intestinal health and IBS symptoms [83].

In addition to clinical studies, in-vitro research has explored the potential effects of vitamin D on the gut–brain axis. One study investigated a nanoformulation of vitamin D3 (VitD3-NS), which was developed to enhance its bioavailability and biological activity [84]. In intestinal cell models, VitD3-NS restored cell viability and reduced oxidative stress in CaCo-2 cells subjected to LPS-induced damage, a model used to mimic IBS-related intestinal dysfunction [84]. VitD3-NS also improved the integrity of tight junctions (TJ) in this model, increasing the expression of TJ proteins (claudin-4, occludin, and ZO-1) and reducing intestinal permeability [84].

While the study also assessed VitD3-NS in neuronal cell models, the methods used to simulate the gut–brain axis remain unclear. The study suggested that VitD3-NS may exert neuroprotective effects by reducing oxidative stress, lipid peroxidation, and markers of neurodegeneration (e.g., phosphorylated tau and amyloid precursor protein), while increasing brain-derived neurotrophic factor (BDNF) and SIRT-1 levels [84]. Additionally, VitD3-NS was reported to activate key survival pathways (ERK/MAPK and PI3K) in neuronal cells [84]. However, since these findings were derived from cell line models, their direct relevance to in vivo conditions remains uncertain.

Given the limited available data on vitamin D and the gut–brain axis in IBS, further research is required to determine whether vitamin D supplementation or its nanoformulations can influence gut–brain interactions in a clinically meaningful way. Future studies using animal models and human participants are necessary to confirm whether vitamin D can directly impact the gut–brain axis and IBS-related neuroinflammation.

**Figure 3:**
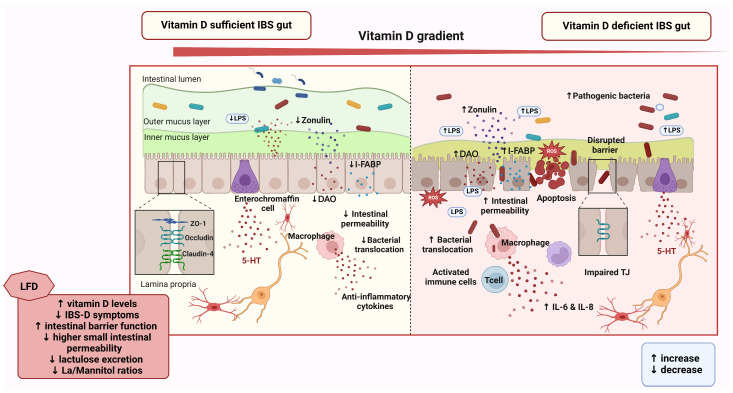
Proposed impact of vitamin D on intestinal barrier integrity in IBS pathophysiology. Figure 3 illustrates the differential effects of sufficient and deficient vitamin D levels on the intestinal barrier and gut–brain axis in IBS. On the left, vitamin D sufficiency is associated with reduced intestinal permeability, lower bacterial translocation, and increased tight junction (TJ) integrity, as evidenced by elevated levels of TJ proteins such as ZO-1, occludin, and claudin-4. Enterochromaffin cells regulate serotonin (5-HT) release, while macrophages produce anti-inflammatory cytokines, contributing to gut homeostasis. On the right, vitamin D deficiency leads to increased zonulin, I-FABP, and DAO levels, resulting in disrupted TJs (including reduced ZO-1 expression), bacterial translocation, and increased IL-6, IL-8, and LPS levels, exacerbating inflammation. Apoptosis and oxidative stress further impair the barrier. The LFD enhances vitamin D levels, decreases IBS-D symptoms, and improves intestinal barrier function by lowering permeability markers (lactulose excretion and La/Mannitol ratio), zonulin, I-FABP, and DAO, further reducing inflammation (IL-6, IL-8) and LPS levels. Together, these findings highlight the potential of vitamin D modulation and dietary interventions in managing IBS-D and gut–brain axis dysfunction. Created with BioRender.com (accessed on 4 March 2025). Abbreviations: IBS, irritable bowel syndrome; LPS, lipopolysaccharides; I-FABP, intestinal-type fatty acid-binding protein; DAO, diamine oxidase; 5-HT, serotonin; ZO-1, zonula occludens-1; IL-6, interleukin-6; TJ, tight junction; LFD, low-fermentable oligosaccharides, disaccharides, monosaccharides, and polyol (FODMAP) diet.

Figure 3 illustrates the differential effects of vitamin D sufficiency and deficiency on intestinal barrier integrity and the gut–brain axis in IBS, highlighting the potential of vitamin D modulation and dietary interventions in managing IBS and gut–brain axis dysfunction.

## 5. Overcoming Methodological Challenges

IBS research has to deal with several methodological challenges. The high heterogeneity that IBS presents complicates patient stratification, and reliance on self-reported symptoms introduces bias. Variability in microbiome analysis methods and study designs also limits reproducibility. Many randomized controlled trials (RCTs) suffer from small sample sizes. Similarly, the current review faces limitations, including the heterogeneity in the available literature, with studies differing in diagnostic criteria, patient populations, and methodologies. The reliance on observational and meta-analytic data may introduce biases, limiting causal conclusions. Future research should prioritize well-powered, standardized studies and explore artificial intelligence (AI) and machine learning to improve predictive modeling and patient stratification. Addressing these challenges and leveraging emerging technologies will strengthen our understanding of IBS and facilitate the development of personalized treatment strategies.

## 6. Conclusions

Vitamin D appears to play a multifaceted role in the gastrointestinal system, and its deficiency may be a factor in both the development and exacerbation of IBS. This review highlights the potential role of vitamin D in the pathophysiology and management of IBS. Emerging evidence suggests that vitamin D deficiency is prevalent among IBS patients and may contribute to the disorder’s molecular mechanisms, including immune dysregulation, gut microbiota imbalance, intestinal permeability, and serotonergic signaling. However, an important unresolved question is whether vitamin D deficiency in IBS patients is a cause or a consequence of the disease. On one hand, the enhanced gut permeability and impaired intestinal function observed in IBS suggest that vitamin D deficiency may be a consequence of malabsorption. This is supported by studies showing that interventions like the low-FODMAP diet (LFD), which improve gut barrier function, also lead to increased vitamin D levels in patients. On the other hand, there is strong biological plausibility for vitamin D deficiency playing a causal role in IBS pathophysiology. Vitamin D has immunomodulatory and anti-inflammatory effects and is crucial for maintaining gut barrier integrity. Deficiency could exacerbate inflammation, disrupt gut microbiota balance, and impair intestinal barrier function, all of which are key factors in IBS. Restoring vitamin D sufficiency through supplementation has shown promise in improving IBS symptoms, QoL, and mental health outcomes, particularly in individuals with baseline vitamin D deficiency, suggesting a potential positive feedback loop where adequate vitamin D levels contribute to gut health, which in turn supports better vitamin D absorption and utilization.

However, the results across studies are inconsistent, with some meta-analyses reporting significant benefits, while others find limited or no effect. This variability underscores the need for further high-quality, randomized controlled trials to clarify the therapeutic potential of vitamin D in IBS, determine optimal dosing strategies, and identify specific patient subgroups that may benefit the most. Additionally, the potential role of the VDR and its polymorphisms in shaping the effectiveness of vitamin D supplementation in IBS is an area of growing interest. VDR, expressed throughout the body, including the gut and brain, mediates vitamin D’s influence on immune regulation, epithelial integrity, motility, and neurotransmitter synthesis. Specific VDR gene variants may contribute to individual variability in response to vitamin D, potentially affecting serotonin levels and gut motility. These genetic factors could help explain why some IBS patients experience significant improvements from vitamin D supplementation, while others show limited benefit. Future studies exploring VDR polymorphisms in IBS could lead to more personalized treatment strategies, optimizing the therapeutic benefits of vitamin D and offering novel insights into IBS management. Comprehending the complex relationship between vitamin D and IBS could pave the way for novel, targeted interventions, ultimately improving the management and QoL for individuals suffering from this challenging condition.

## Figures and Tables

**Table 1:** Summary of meta-analyses assessing the role of vitamin D supplementation on IBS symptom severity and Quality of Life.

Meta-Analysis	Date	No. of Studies	No. of Participants	Comparing Arms	Effect Size	95% CI	*p* Value	I^2^ (%)	Effect Size	95% CI	*p* Value	I^2^ (%)
					Severity of Symptoms				Quality of Life			
Abuelazm et al. [48]	2022	6	616	Vitamin D vs. placebo in IBS	−45.82 (MD)	[−93.62, 1.98]	0.06	97.07	6.19 (MD)	[0.35, 12.03]	0.04	97.07
Bin et al. [49]	2022	12	1331	Vitamin D vs. placebo in IBS	−0.43 (SMD)	[−0.89, 0.03]	-	85.6	0.65 (SMD)	[0.14, 1.15]	-	85.6
Chong et al. [50]	2022	8	685	Vitamin D vs. placebo in IBS	−0.77 (SMD)	[−1.47, −0.07]	0.04	91	0.54 (SMD)	[−0.34, 1.41]	0.15	87
Huang et al. [51]	2022	4	335	Vitamin D vs. placebo in IBS	−55.55 (WMD)	[−70.22, −40.87]	<0.001	53.7	14.98 (WMD)	[12.06, 17.90]	<0.001	0.0
Cara et al. [52]	2024	12	-	Vitamin D vs. placebo in IBS	−25.89 (MD)	[−55.26, 3.48]	0.15	92.8	3.19 (MD)	[2.14, 4.24]	0.00001	0.0

Abbreviations: No., number; 95% CI, 95% confidence interval; I^2^, heterogeneity; vs., versus; MD, mean difference; SMD, standardized mean difference; WMD, weighted mean difference.
